# The Intraosseous Dysfunction in the Osteopathic Perspective: Mechanisms Implicating the Bone Tissue

**DOI:** 10.7759/cureus.6760

**Published:** 2020-01-24

**Authors:** Eduardo Bicalho

**Affiliations:** 1 Osteopathic Medicine, Colégio Brasileiro De Osteopatia - CBO ( Brazillian College of Osteopathy), Sorocaba/SP, BRA

**Keywords:** osteopathic medicine, osteopathic manipulative therapy, manual therapy, somatic dysfunction, intraosseous dysfunction, sensitization

## Abstract

The somatic dysfunction (SD) is a protagonist in the context of theories and practices involving osteopathy and various other manual therapy methods. It is considered an obstacle to the body's inherent self-regulatory capabilities, and several tissues may be involved in this dysfunctional process, including the bone. The so-called intraosseous dysfunction refers to the restriction of natural flexibility of the fibrous components of the bone tissue matrix, or of the nonossified cartilaginous or membranous areas. Bone is a connective tissue composed of inorganic material and specialized cells organized in a hydrated extracellular matrix that provides the mechanical qualities to the tissue. The development of the bone tissue is a continuous process throughout life, and some bones fuse only years or decades after birth. It has microanatomical continuity with other adjacent structures and its different compartments are supplied by fluids, as well as somatic and autonomic innervation. Several studies show the phenomenon of bone tissue sensitization under traumatic, pathological conditions and also movement restriction. The purpose of the article is to review well-established knowledge and recent scientific findings regarding bone tissue anatomy and physiology, in an attempt to offer insights that could be applied to better understand the mechanisms implicating the intraosseus dysfunctions and its local and global repercussions.

## Introduction and background

The term somatic dysfunction (SD) is defined as “…impaired or altered function of related components of the somatic (body framework) system: skeletal, arthrodial and myofascial structures, and their related vascular, lymphatic, and neural elements” [1]. It is considered a reversible functional disorder, an obstacle to body's inherent self-regulatory capabilities. Initial hypotheses related its genesis to neurological [2] and nociceptive [3-5] imbalances, and more recently to changes in the fascial tissue physiology [6].

The SD can affect any kind of connective tissue [1], including the bone (intraosseous dysfunction) [7], which has important physiological [8] and mechanical [9] characteristics, neurological (somatic and autonomic) [10-11] and fluidic supply [12-14], as well as microanatomic connections with adjacent tissues [15-16]. Generally, only its outer layer, the periosteum, has been considered as part of the fascial system [17]. However, recently, some authors [16-18] defend the proposal that all compartments of the bone tissue deserve to be considered part of this system for reasons described below. The traditional definitions [1] of the SD focus especially on the relation between different anatomical components. However, when bone tissue is inserted in this context [7], the dysfunction can also be pondered within the tissue itself, regarding to its mechanical, fluidic, and neurological impacts that might be produced locally and globally.

## Review

Bone tissue composition

Bone is a highly vascularized and innervated connective tissue composed of cells specialized in synthesis (osteoblasts) and degradation (osteoclasts), and also in mechanosensory functions (osteocytes) [8]. These cells promote constant remodeling of bone tissue throughout life according to functional demand and are disposed in a hydrated mineralized extracellular matrix that determines the mechanical qualities of bone, being formed by organic materials (collagen, and proteins such as proteoglycans and glycoproteins) that allow flexibility, and inorganic materials (calcium and phosphate) that provide resistance to bone tissue [19]. Collagen has thixotropic and piezoelectric properties that permit continuous adaptation to the mechanical stresses imposed on the tissues. Bones have an outer (periosteum) and inner (endosteal) lining. The periosteum is formed by dense connective tissue that surrounds the outside of the bone, except the joints. Protects, nourishes, and assists in bone formation and fracture repair. The endosteum covers the inner layer of the cortical bone, and is composed of loose connective tissue [20]. Sharpey's collagen fibers connect the periosteum to the innermost cortical regions of the bone until it reaches the endosteum, especially in areas exposed to increased mechanical stress. These fibers provide important microanatomic continuity between bone tissue components and their envelopes [15].

Mechanical properties

Bone is composed of viscoelastic tissue that is very capable of adapting to its physiological and mechanical environment, so that it can perform several physiological purposes, including protecting vital organs and serving as a mechanical lever for muscle contractions [9]. Although it is one of the most rigid structures in the human body, bone tissue deforms during body movements and also during trauma imposed on it. Some important conditions that directly influence the mechanical properties of bone are highlighted [21]: its deformation is not proportional to the load imposed on it; its mechanical properties vary according to the pace of the load; its mechanical behavior is dependent on the fluid present in bone tissue; bone is composed of different types of bone tissue with different mechanical properties; its mechanical properties are not identical in all directions; it is in constant remodeling and has different properties in different periods of life. Mineralized matrix components promote strength, and collagen fibers that are resistant to tension and traction ensure the flexibility of bone tissue and supply its energy absorption capacity. Thus, changes in collagen property directly affect the mechanical quality of bone by increasing its susceptibility to fractures [9].

 Osteogenesis

The process of ossification or osteogenesis occurs in two ways: intramembranous - some bones of the cranial vault, clavicle, maxilla and mandible, by the deposition of bone in the primitive connective tissue (mesenchyme) especially in the so-called ossification centers; and endochondral - long bones (growth plates), some bones at the cranial base, the sacrum and sternum, where cartilage acts as a precursor in the synchondrosis regions [11]. Synchondroses are cartilaginous joints that allow small movements between bones. Usually temporary and exist during skeletal development until bone maturation occurs and cartilage between bones becomes thinner and then replaced with bone tissue [22]. After birth some bones are divided into distinct parts that are joined by cartilaginous tissue, and the complete fusion process between these parts occurs during life. Table 1 shows some examples:

**Table 1:** Period of fusion after birth of some human bones.

Bone	Divisions	Time of fusion
Sacrum	Five vertebras	25-30 years [23]
Iliac bone	Ilium, ischium, and pubis	11-17 years [23]
Sternum	Esternebras of the sternum body	5-25 years [24]; 4 years-puberty [23]
	Xifoesternal joint	About 35 years [23]
Frontal	Metopic suture	9 months [25]
Occipital	Posterior intraoccipital synchondrosis (between squama and lateral parts)	1-5 years [26]; 1-4 years [23]
	Anterior intraoccipital synchondrosis (between lateral parts and basilar portion)	3-7 years [23,26]
Sphenoid	Pre- and post-sphenoid	1 year [23,27]
Temporal	Petromastoid and scamotympanic portion	1 year [23]
Mandible	Mentonian symphysis	1 year [23]
Spheno-occipital synchondrosis		13-17 years [28]
Spheno-etmoidal synchondrosis		6 years [22]
Humerus	Proximal epiphysis	12-20 years [29], 14-21 years [23]
	Distal epiphysis	11-19 years [29], 11-18 years [23]
Femur	Proximal epiphysis	11-19 years [29], 12-19 years [23]
	Distal epiphysis	14-19 years [29], 14-20 years [23]

Bone remodeling is a continuous process throughout life. Its formation increases in areas that receive greater stress, and its absorption increases in regions of disuse [11]. For a mechanical stimulus to be transformed into a biological signal (mechanotransduction) in bone tissue, it is necessary that the tensions project on the cell membrane proteins (integrins) modifying their spatial structure. Osteocytes play a fundamental role in mechanotransduction that drives bone remodeling according to mechanical demand [30]. Mechanical stress, hydrostatic pressure, and especially fluid flow, serve as stimuli for osteocytes, which mediate bone formation and absorption activities promoted by osteoblasts and osteoclasts, respectively [30].

Bone cells have short- and long-term memory capacity, such as habituation (reduced cell transduction after repetitive stimuli) and sensitization (increased cellular response to a given stimulus), in such a way that the bone tissue adjusts according to the demand imposed on it [31]. Robling and colleagues [32] demonstrated the phenomenon of mechanical habituation or desensitization of bone tissue, highlighting the need for recovery periods between functional demands to restore osteogenic and mechanosensory capacity of bone tissue.

Fluidic supply

 Apparently under normal conditions, there are two types of fluids within the bone: blood (venous and arterial) and interstitial fluid [12]. The rich arterial and venous vascularization of bone tissue has several functions aimed at homeostasis of the skeletal system, such as supplying osteogenesis-linked cells and also bone marrow hematopoietic stem cells [13]. The dynamics of bone tissue interstitial fluids plays an important role in its mechanosensory system, as bone deformation promotes changes in interstitial flow that are detected by osteocytes. Skeletal and cardiac muscle contractions also influence the interstitial flow mechanism. This mechanism is fundamental in the physiology of bone formation and remodeling according to functional demand [12].

The existence of lymphatic vessels in bone tissue is still doubtful, as attempts to demonstrate their presence in bone and marrow tissue have not been successful [12]. Edwards and colleagues [14] observed, through immunohistochemical analysis, the presence of lymphatic vessels only in the periosteum region of healthy bones. In some cases of bone sarcoma analyzed, the connective tissues surrounding the tumor had lymphatic vessels. However, Shi and colleagues [33], using immunofluorescence analysis, observed the presence of lymphatic tissue in various soft tissues of joints and in the periosteum of long bones of rats. Their method of analysis allowed to observe occasionally (in about 10% of the sample) lymphatic vessels in cortical bone and bone marrow.

Bone innervation

All bone compartments have somatic and sympathetic sensory innervations; however, nerve fiber density is variable - for every 100 fibers in the periosteum, two in the bone marrow, and 0.1 in the mineralized bone [34]. Sensory receptors of bone tissue are able to detect mechanical, inflammatory, and nociceptive stimuli. The regions of mineralized bone that are most subjected to stress and mechanical loads, are the most vascularized and have the highest density of sensory and sympathetic fibers. Small sympathetic fibers in the periosteum pass through the cortical bone following the vessels of the Volkmann and Havers channels, controlling vasomotricity [11]. Bjurholm and colleagues [35] showed the presence of calcitonin gene-related peptide (CGRP) and substance P immunoreactive nerve fibers, pertinent to nociceptive activity and vasodilation, not only in the periosteum, but also within the compact bone and bone marrow in rats. Somatic and sympathetic nerve fibers immunoreactive to substance P and CGRP were also found penetrating the vessels in the Volkmann channels and the periosteum of the vault and mandible of adult rats. In the tibia, nerve fibers are more concentrated in the regions of the epiphyses, suggesting greater neural density due to increased bone metabolic activity in this area during growth [36]. In addition to the presence of primary afferent nerve fibers containing CGRP and substance P, sympathetic fibers containing vasoactive intestinal peptide (VIP) were also found in the mandible periosteum of rats. It is suggested that these sympathetic fibers may be related to osteoclast bone absorption activity [37].

In addition to somatic and sympathetic innervations, cholinergic parasympathetic fibers have also been detected in bone tissue, but their density and pattern of bone tissue innervation are unknown. Parasympathetic activity also appears to be directly linked to bone metabolism, being antagonistic to sympathetic activity thus favoring bone mass accumulation, inhibiting osteoclast activity [38]. Bone marrow has parasympathetic fibers, but the autonomic function in this area is unknown. The high concentration of nerve fibers suggests that in addition to vasomotor activity, autonomic innervation directly participates in hematopoiesis, controlling the release of bone marrow cells into the peripheral circulation [39]. One study showed the influence of the circadian cycle on bone remodeling, with absorption peaking during the day with dominant sympathetic activity, and at night bone formation is more active due to the predominance of parasympathetic activity [40].

Nociception of the bone tissue

It is well established in the scientific medical world that several conditions can cause bone pain, such as fractures, osteoarthritis, rheumatoid arthritis, low back pain, bone cancer, and also various genetic diseases that affect bones and joints [34]. Bone tissue has thin myelinated (A-delta) and unmyelinated (C) fibers responsive to harmful chemical and mechanical stimuli that participate in inflammatory pain signaling. These fibers respond to harmful high-threshold mechanical stimuli caused by disorders related to increased intraosseous pressure [10]. When a bone is fractured, for example, its repair process is made in distinct, properly recognized phases. During the inflammatory phase, allodynia (when a non-nociceptive stimulus causes pain perception) and hyperalgesia (when a nociceptive stimulus causes excessive pain sensation) may occur due to the release of cytokines, histamine, bradykinin, etc. [41]. When the inflammatory process causes the increase in intraosseous pressure to exceed a certain level, the fluids that permeate the bone can penetrate the subperiosteal space and induce pain [41]. Neurogenesis related to ectopic sprouting of nerve fibers may also be involved in bone nociception. Following bone injury or pathology, several neurotrophic factors are released, inducing sprouting and increased innervation of the bone marrow, mineralized bone, and periosteum [34]. This occurs even in a fracture that is normally healing in the callus region so that it is protected until complete repair occurs. When this happens, the callus is reabsorbed and this innervation returns to its natural state. However, in cases of bone disease or injury, in which the repair process does not take place properly, this nerve sprouting may not return to its normal state, keeping the bone hyperinervated in such a way that non-noxious mechanical loads will be perceived as nociceptive events [34].

Can bone tissue be inserted in the fascial continuum?

The term fascia has been widely used nowadays, but it can be assumed that there is still a large inconsistency about its definition. It has recently been proposed that the fascial system: “...interpenetrates and surrounds all organs, muscles, bones and nerve fibers, endowing the body with a functional structure, and providing an environment that enables all body systems to operate in an integrated manner. It incorporates elements such as adipose tissue, adventitiae and neurovascular sheaths, aponeuroses, deep and superficial fasciae, epineurium, joint capsules, ligaments, membranes, meninges, myofascial expansions, periostea, retinacula, septa, tendons, visceral fasciae, and all the intramuscular and intermuscular connective tissues including endo-/peri-/epimysium” [17]. Regarding the bone tissue, most of the definitions usually consider only the periosteum as part of the fascial system. However, for Sharkey [16], definitions that do not include the other components of bone tissue as part of the fascial system are incomplete, as bone is a vital element for musculoskeletal continuity, and there is also a close embryological relationship between bones and the fascial tissue. The author [16] points out that the concept of continuity of the fascial system also deserves due importance when looking at the connection of ligaments and tendons in the periosteum, which in turn connects to the bone matrix through Sharpey fibers. Bordoni and Lagana [18], on the other hand, proposed that bone tissue corresponds perfectly as part of the fascial system, taking into account some relevant aspects such as the microscopic anatomical continuity of tissues, the embryological origin of bone and fascial tissue, the autocrine and paracrine tissue activities influencing itself and other body structures and systems, and the mechanometabolic responses of bone cells. From these observations, these authors [18] describe a new definition of the fascial system, which includes liquid and solid structures (such as bone), and adds features to the fascial tissue such as the ability to support, divide, penetrate, nourish and connect all structures of the body.

The somatic dysfunction

Several models have been proposed to explain the genesis of the SD and its repercussions. Korr [2] suggested that an afferent bombardment originating on proprioceptors causes a "facilitation" or reduction of the activation threshold of spinal cord interneurons, enhancing the sensory, motor, and sympathetic activity of the involved segment. Van Buskirk [3] emphasized the primordial participation of nociceptors in a neurogenic inflammation arising from stress (mechanical, chemical, thermal) in initiating the process of constant afferences to the spinal segment. Howell and Willard [4] extended the proposal of the nociceptive model, describing how a noxious stimulus originating in a primary afferent nociceptor is conducted through an afferent neuron to the posterior horn of the medulla producing neurogenic inflammation (central sensitization), that may lead to antidromic action potentials that support peripheral inflammatory reactions, also facilitating peripheral receptors (peripheral sensitization). These sensitization events might also involve altered activity in the anterior root fibers (somatic and sympathetic) of the spinal cord causing changes in skeletal muscle tone, vasomotor, sudomotor, and visceral activity of the affected segments. Tozzi [6] published an extensive evidence-based literature review and introduced the neurofasciogenic model of the SD from the perspective of aggregating neurological influences in a multidimensional perspective. For the author [6], in addition to the neurophysiological influences described in other models, SDs have in their genesis fundamental relationships with modifications in specific properties of the fascia, such as its architecture, contractility, viscoelasticity, fluid content and dynamics, pH, autonomic and somatic neural interactions, metabolic influences, piezoelectricity, and epigenetics.

Palpatory sensitivity (allodynia or hyperalgesia) of a dysfunctional tissue is well established as one of the parameters that evidence a SD [5], and it is a fundamental condition that may reveal a local process or neurological reflex, possibly related to the phenomenon of central sensitization [42].

Central sensitization

When a tissue stress event occurs (injury, inflammation), inflammatory mediators are released locally, triggering the activity of nociceptors and neurotransmitters that carry these afferences to the spinal cord through fine-caliber neurons (initially) [4]. The duration and extent of this nociceptive activity will depend on the nature of the damage done. Neurotransmitters reach the dorsal root ganglion of the sensory neuron, and even before reaching the spinal cord where they promote neurogenic inflammation, a reflex occurs (dorsal root reflex) that promotes the antidromic flow of neurotransmitters back to the peripheral receptor [43]. When nociceptive activity is potent enough or sustained over time, it can maintain the alert state of specific spinal cord neurons, the wide dynamic range (WDR) neurons, making them sensitized. This phenomenon occurs when harmful stimuli in receptors located in any sensory innervated tissue maintained over time promote neuroplasticity events with increased excitability of neurons involved in central nociceptive activity and in the higher centers responsible for pain perception [43]. Peripheral receptors maintain sensitized such that their activation thresholds become reduced. Large-caliber neurons, which conduct mechanical stimuli under physiological conditions, also lead to conduct nociceptive stimuli. The consequences of this process are the conditions of allodynia and/or hyperalgesia [44]. This causes peripheral stimuli to have their efferent responses amplified. This process of exaggerating pain perception to a stimulus may last beyond the trigger factor caused by injury or inflammation of a peripheral tissue, such that it is maintained by superior central nervous system (CNS) activities [43].

Central sensitization can cause receptors from other tissues to be affected as well, when their neurons converge on the same affected spinal cord segment through WDRs that are overexcited by the influence of pain-mediating substances such as glutamate and substance P. Lai and colleagues [45] showed that the skin of the suprapubic region becomes sensitive to pressure in individuals with urinary tract infection; and the research made by Sarkar et al. [46] showed that inflammation of the lower esophageal area caused sensitization of uninjured areas of the same organ by reflex as they are innervated by the same spinal cord segment; and also that sensitization of a peripheral receptor may perpetuate over time even if the stress event (injury, inflammation) has been resolved by a process sustained by the upper centers of the CNS. The sensitization process is reversible and can be modulated [43], and under normal conditions, when tissue repair occurs, nociceptive bombardment is ceased and the phenomenon resolves spontaneously. Some important questions related to this process are still not totally clear: why does sensitization resolve spontaneously in most cases but may become persistent in others? Is the intensity or duration of nociceptive activity directly related to process maintenance?

Bone tissue sensitization

Pathologies affecting bone tissue may cause allodynia or hyperalgesia of the skin near the lesion or even in distant regions (secondary hyperalgesia) reflecting sensitization of cutaneous afferent neurons or their central projections. It is clearly known that in the CNS, dorsal root neurons can be activated and sensitized by bone tissue [10]. When a fracture occurs, the presence of allodynia when using the affected limb, as well as mechanical and thermal hyperalgesia, suggests that the phenomena of peripheral and central sensitization are also present in this condition. The sprouting of CGRP-reactive nerve fibers and substance P suggests that one of the possible mechanisms involved in peripheral sensitization in bone pain caused by fractures is increased neuropeptidergic neurotransmission. Li and colleagues [47] observed that the rat tibial fracture immobilized for four weeks triggered the activity of afferent C fibers with substance P release in the spinal cord posterior horn, resulting in chronic glial activation and central sensitization. When rapid and effective repair of an injury or stress to bone tissue occurs, sensitization-related events usually return to pre-injury levels. However, when this condition of resolution does not occur normally, multimodal bone tissue receptors may remain active, alert, and sensitization may perpetuate and accentuate their intensity causing persistent bone pain [34].

In addition to the pathological or traumatic conditions that promote bone tissue sensitization, the work of Guo and colleagues [48] showed that immobilization also promotes local tissue changes similar to those occurring when there is bone fracture (e.g. allodynia, edema, cutaneous inflammatory mediators), in afferent sensory neurons (substance P, CGRP) and in the spinal cord (inflammatory mediators). The mobility restriction experimentally produced in this research led to peripheral sensitization of bone tissue, as well as evidence of neurogenic inflammation involving afferent neurons and the CNS (spinal cord). These findings deserve importance when considering changes in bone tissue mobility and neurological consequences.

Intraosseous dysfunction: possible mechanisms and repercussions 

The so-called intraosseous dysfunction is the restriction of the natural flexibility of the fibrous components of the bone tissue matrix, or of the nonossified cartilaginous or membranous areas [7]. Traumas or strains that produce restrictions on cartilage joints that have not yet fused can provide focus of tension and asymmetries during bone development [7]. Considerations about intraosseous dysfunctions are traditional in pediatric osteopathic field, especially in conditions of cranial plagiocephaly [7]. Recent research [49-50] has shown significant responses of the manual approach under these conditions.

In addition to bone asymmetries caused by intraosseous dysfunctions that may occur in the early stages of life, other mechanisms described above could apparently be involved both in the genesis and in the consequences of intraosseous dysfunctions [43-44, 47-48]. It is proposed to take into consideration these mechanisms when assessing the globality and complexity of individuals. The intraosseous dysfunctions can affect any bone in the body [7] and it is proposed the need for its manual approach is not only in infants and children, but also in adults, due to the constant remodeling of bone tissue throughout life [8]. From a neurological perspective, any tissue that has sensory innervation can become a primary source of aberrant afferent stimuli involved in the sensitization phenomena [4, 44]. Locally, allodynia or primary hyperalgesia may occur in bone tissue [47-48], just as afferent bombardment may promote secondary consequences (secondary hyperalgesia) in other tissues innervated by corresponding neurological levels [45-46]. Additionally, the persistence of neural sprouting at the site of a bone lesion may maintain tissue hyperinervation [34] causing aberrant afferences to the nervous system to be sustained.

Evidences also support that bone tissue immobilization may also play a role in the condition of peripheral and central sensitization [48]. In other words, the restriction of mobility generates not only mechanical, but also local and central neurological consequences. Constant afferences triggered primarily by stress or loss of mobility in bone tissue could also cause efferent autonomic reflexes, which secondarily could alter vasomotor activity [42]. Conversely, it is also presumed to recognize the secondary consequences to bone tissue, when constant afferent stimuli provoked by another tissue (through the WDR neurons) may promote vasomotor changes that affect bone components [45].

The possibility to consider the bone tissue as part or as an expansion of the fascial system [16,18] may suggest a reflection on the influence of intraosseous dysfunctions and the possible repercussions on the mechanical quality of the bone itself in its local and global biomechanical adaptation to movements [9]. It is also possible to consider the possible impact that the propagation of these mechanical tensions initiated in the bone tissue promote on adjacent fascias [16] that cover, permeate and connect other structures, and also the repercussions of theses tensions on the whole body, bearing in mind the biotensegrity model. Considering aspects related to the genesis of SD proposed by the neurofasciogenic model [6], such as changes in physiological mechanisms of the fascia as their viscoelastic and piezoelectric capacities, and fluid dynamics, it is plausible to consider the effects of such repercussions also on bone tissue affecting its physiological mechanisms. Following this thought, the dysfunctions could negatively affect the fluid dynamics [12] of bone tissue and consequently its mechanosensory activities directly linked to bone metabolism and the mechanical qualities of the organic components of the bone matrix [19].

## Conclusions

Based on the anatomical and physiological characteristics of bone tissue described above, it is proposed to consider some relevant conditions and repercussions regarding intraosseous dysfunctions. It is possible to ponder the impact of the dysfunctions affecting bones that fuse after birth, and the possible consequences on the development, morphology, fluid dynamics, and mechanical quality of bone tissue. However, it is also plausible to reflect on the possible participation of the intraosseous dysfunction in the mechanisms of peripheral and central sensitization, as well as the repercussions of these processes on the physiological mechanisms of bone tissue itself, and also of all the neurologically interconnected tissues. Finally, it is proposed to consider intraosseous dysfunctions from mechanical, neural and fluidic perspectives, when an individual is fully evaluated at any moment in life.
